# The existence of bronchiectasis predicts worse prognosis in patients with COPD

**DOI:** 10.1038/srep10961

**Published:** 2015-06-16

**Authors:** Bei Mao, Hai-Wen Lu, Man-Hui Li, Li-Chao Fan, Jia-Wei Yang, Xia-Yi Miao, Jin-Fu Xu

**Affiliations:** 1 Department of Respiratory and Critical Care Medicine, Shanghai Pulmonary Hospital, Tongji University School of Medicine, No. 507 Zhengmin Road, Shanghai, China; 2 Soochow University, Suzhou, China

## Abstract

Bronchiectasis is prevalent in patients with COPD. The objective of this study was to assess the clinical characteristics and prognostic value of bronchiectasis in patients with COPD in China. Data from patients diagnosed with COPD at the Shanghai Pulmonary Hospital between January 2009 and December 2013 were retrospectively collected and analyzed. SPSS statistical software was used to analyze the data. Data from 896 patients with COPD were analyzed. Bronchiectasis was present in 311 patients. The isolation of pseudomonas aeruginosa (PA) from sputum was the variable most significantly associated with the presence of bronchiectasis in patients with COPD (hazard ratio (HR), 2.93; 95% confidence interval (CI), 1.35–6.37; P = 0.007). During follow-up (median of 21 months; interquartile range: 10-39 months), there were 75 deaths, of which 39 were in the bronchiectasis group. The presence of bronchiectasis (HR, 1.77; 95% CI, 1.02–3.08; P = 0.043) was associated with an increase in all-cause mortality in patients with COPD. These results suggest that bronchiectasis in patients with COPD was associated with the isolation of PA from the sputum. Bronchiectasis was an independent risk factor for all-cause mortality in patients with COPD.

Chronic obstructive pulmonary disease (COPD) is the most common respiratory disease worldwide. The COPD International Patient Survey, which involved 12 countries, showed rates of estimated COPD prevalence that were similar across countries and higher than those reported a decade ago^1^,^2^,^3^,^4^. The increase in COPD prevalence and mortality has led to a greater economic burden of disease for patients and their families^5^,^6^,^7^,^8^.

Previous studies have reported a very high prevalence of bronchiectasis in patients with moderate to severe COPD^9^,^10^,^11^. The diagnosis and treatment of COPD and bronchiectasis together seems to be more complicated than the diagnosis and treatment of either condition alone. Current research indicates that patients with COPD and bronchiectasis have more serious bronchial inflammation, longer and more intense exacerbations, more frequent colonization of the bronchial mucosa by potentially pathogenic microorganisms (PPM), and a higher degree of functional impairment^10^,^12^. For this reason, the early diagnosis and treatment of bronchiectasis is very important for patients who have been diagnosed with severe COPD. However, few studies have reported on the clinically predictive factors of bronchiectasis in patients with COPD. Therefore, the objective of this study was to identify the relevant factors and prognostic value of bronchiectasis by collecting clinical and follow-up data from patients diagnosed with COPD in a specialized hospital in Shanghai, China.

## Methods

### Study Subjects

The study retrospectively collected the data from 896 consecutive inpatients diagnosed with COPD between January 2009 and December 2013 in the Shanghai Pulmonary Hospital. Patients who had not received a chest HRCT scan examination and patients with indecipherable HRCT scan images were excluded. Clinical physicians who were involved in the study collected all of the data. Written informed consent was obtained from all of the patients. The Ethics Committee of Shanghai Pulmonary Hospital approved the study protocol (K14-157). All aspects of the study were performed in accordance with relevant guidelines and regulations.

### Diagnosis of COPD and Bronchiectasis

COPD was diagnosed as the presence of a post-bronchodilator forced expiratory volume in 1 second/ forced vital capacity (FEV_1_/FVC) <70% in patients with a long smoking history, according to the criteria published by the Global Initiative for Chronic Obstructive Lung Disease (GOLD)^13^. An exacerbation of COPD is an acute event characterized by a worsening of the patient’s respiratory symptoms that is beyond normal day-to-day variations and that leads to a change in medication^13^,^14^,^15^,^16^.

A chest HRCT scan was used to confirm the diagnosis of bronchiectasis. High-resolution images were obtained in full inspiration at 1-mm collimation and 10-mm intervals from the apex to the base of the lungs. The presence of bronchiectasis was based on the following criteria published by Naidich and colleagues: (1) lack of tapering of the bronchi, (2) dilation of the bronchi when the internal diameter was larger than that of the adjacent pulmonary artery, or (3) visualization of the peripheral bronchi within 1 cm of the costal pleural surface or the adjacent mediastinal pleural surface^12^,^17^. The type of bronchiectasis was defined according to the morphology of bronchiectasis. We used a hierarchical diagnosis system to determine the final diagnosis of bronchiectasis. To limit inter-rater variability, two radiologists and a respiratory physician independently interpreted the HRCT scan of each patient. If there was divergence in a diagnosis, the three professionals discussed the case and made a final decision. Cases of small bronchiectasis only visible in a single pulmonary segment were ignored, because this sign can appear in a large proportion of the healthy population^18^.

### Interview Questionnaire and Blood Samples

**Figure 1:**
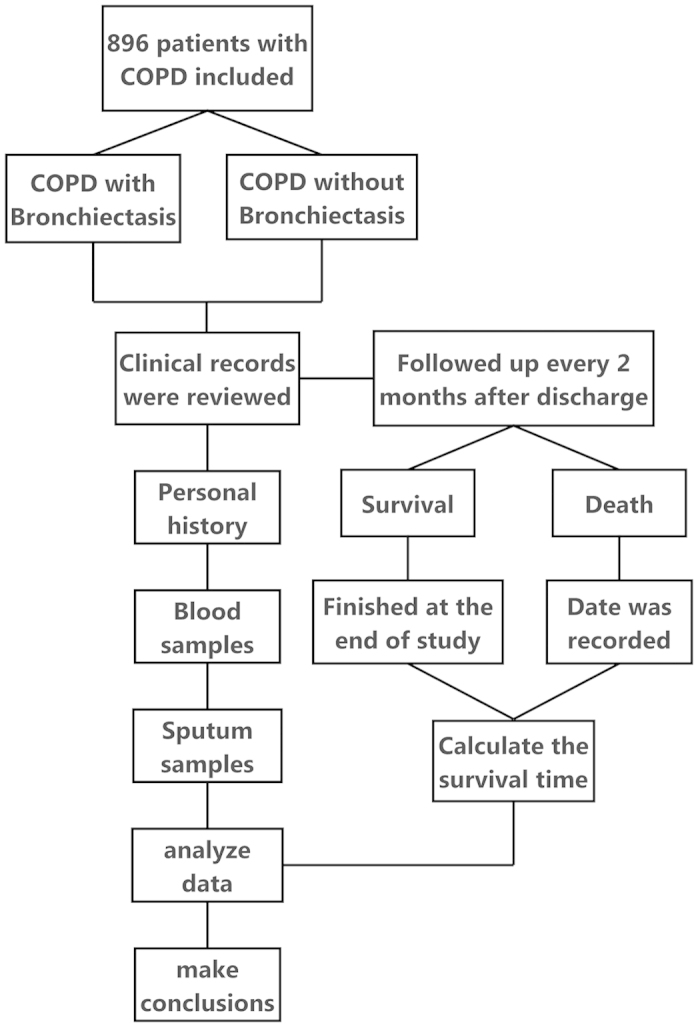
The detailed study procedure to ensure that all of the researchers performed their assignments according to protocol.

A standardized protocol was used for obtaining informed consent from each subject during a medical visit. The interview questionnaire that was used included questions on the following topics: general and anthropometric information (i.e., age, sex, and body mass index); smoking history (where the smoking index was defined as the number of cigarettes smoked per day multiplied by the number of years smoked); and history of respiratory illness (i.e., nasosinusitis, tuberculosis (TB), and pneumonia) and clinical manifestation (e.g., the onset of symptoms, the property of chronic expectoration, the presence of wheezing, etc.). In addition, C-reactive protein (CRP) level and erythrocyte sedimentation rate (ESR) were used as markers of systemic inflammation and albumin level was used as a marker of nutritional status. Hemoglobin level and oxygen and carbon dioxide partial pressures (PO_2_ and PCO_2_, respectively) were also assessed. Finally, blood glucose level was also collected because recent research has found this variable to be correlated with COPD mortality^19^,^20^,^21^,^22^. The detailed study procedure is shown in Fig. 1.

### Sputum Samples

The microbiologic analysis of daily spontaneous morning sputum was requested for each patient during hospitalization. Patients were taught how to correctly collect sputum samples using the most sterile technique possible, and they were asked to deposit the samples at the hospital laboratory within a maximum of 3 h after collection. Sputum samples were accepted if they contained fewer than 10 squamous epithelial cells and more than 25 leukocytes per low-powered field. The samples were separated from saliva, Gram stained, and homogenized. Diluted secretions were plated on blood, chocolate, MacConkey agar and Sabouraud agar. Sputum cultures were expressed as colony-forming units (CFUs) per mL. Based on previously published methods, a cutoff point of 10^3^ CFUs/mL or more was defined as significant for the identification of abnormal positive culture results for PPM^23^,^24^,^25^. Isolated bacterial agents were classified into PPM strains, including PA, *Klebsiella pneumonia, Escherichia coli, Baumannii, Enterobacter cloacae, Aspergillus, Monilia albicans* and other pathogenic microorganisms. The microorganism isolation tests were performed by laboratory technicians who are blind to the clinical characteristics of the subjects in the study.

### Survival Analysis

In our department, all of the patients with COPD were routinely asked to sign a consent form when they were admitted to the hospital. Patients signed the consent form to authorize follow-up every 2 months through telephone or face-to-face interviews. The follow-up was completed on May 31, 2014. A patient was considered lost to follow-up if we were unable to contact him/her at each follow-up session during the study period. The endpoint of this study was all-cause mortality. Information regarding the cause and date of death was obtained from hospital medical records if the patient died in the hospital or from official death certificates in other situations.

### Statistics

The statistical package SPSS, version 19.0 (SPSS, Chicago, Illinois), was used for the statistical analysis. The data were tabulated as the mean and standard deviation in the case of quantitative variables and as absolute numbers and percentages in the case of qualitative variables. The Kolmogorov-Smirnov test was used to analyze the distribution of variables. The patients were divided into the following two groups: patients who had bronchiectasis and patients who did not have bronchiectasis. In the bivariate analysis, the Student t test for independent variables was used to analyze variables that were normally distributed and the Mann-Whitney U test was used to analyze variables that were non-normally distributed. Qualitative variables were compared using the chi-square test. In multiple comparisons, quantitative variables were compared using one-way ANOVA. If a variable was found to be significant, then the SNK-q test was used to analyze the comparison among groups in multiple comparisons. Depending on whether variables were normally or non-normally distributed, either a Spearman or a Pearson coefficient was calculated to assess the correlation between variables. In the case of elevated collinearity between two variables (Spearman correlation test > 0.6), the variable with greater clinical significance was chosen according to the authors’ judgment. A logistic regression model was used to determine the factors that were independently associated with the presence of bronchiectasis. A Cox proportional hazard regression model was used to assess the factors associated with survival. The variables that presented statistically significant differences (P < 0.05) in the bivariate analysis and were of clinical interest were included as the independent variables in the first model. Then, the forward stepwise technique (i.e., the Wald test) was used to remove the variables that had a P > 0.1 from the final model. Bronchiectasis and all-cause mortality were the dependent variables. Survival curves for the two groups (i.e., COPD patients with and without bronchiectasis) were constructed according to the Kaplan-Meier method and were compared using the log-rank test. HR and 95% CI for the independent variables were also calculated. P ≤ 0.05 was considered to be significant.

## Results

A total of 896 patients (mean age 66.2 [9.6] years; 84.9% men) with COPD were included in the final analyses after ten patients who did not have HRCT scans and one patient who had uninterpretable HRCT results were excluded. Bronchiectasis was present in 311 (34.7%) patients. Sixteen patients presented a history of nasosinusitis (1.8%), 108 patients presented a history of tuberculosis (12.1%), and nine patients presented a history of at least one pneumonia episode (1.0%). No other conditions that can trigger bronchiectasis were found in our patients (e.g., a1-antitrypsin deficiency, significant immunodeficiencies, systemic diseases, high-risk professions [72% were retired], etc.). An average of three valid sputum samples were collected from patients who had daily sputum production during the study. The most frequently isolated PPM in the entire patient population was *Monilia albicans* (245 patients). *Aspergillus* was isolated in 40 patients. PA was isolated in 38 patients, 27 of whom had bronchiectasis. *Klebsiella pneumoniae* was isolated in 26 patients. Mycobacterium was detected in 19 patients, nontuberculosis mycobacteria (NTM) in 8 patients and mycobacterium tuberculosis in 11 patients. No samples or unqualified samples were collected from 170 patients (19%).

The differential characteristics of the group with bronchiectasis(n = 311) and the group without bronchiectasis (n = 585) are shown in Table 1 and Table 2. Patients with COPD and bronchiectasis tended to be older and male, and they had a longer duration of symptoms, more purulent sputum expectoration, greater systemic inflammation, poorer nutritional status, a longer length of hospitalization, and more positive cultures of PPMs.

The characteristics of the different types of bronchiectasis that patients had during the study are described in Table 3. Of the patients, 256 (82.3%) had cylindrical bronchiectasis. These patients presented with higher levels of PO_2_. The severity of dyspnea in patients with cylindrical bronchiectasis was moderate compared with that in patients with cystic or mixed bronchiectasis. However, there was no significant difference in the severity of dyspnea between patients with cystic bronchiectasis and patients with mixed bronchiectasis (P = 0.713).

The results of the logistic regression, which included the final four variables that were chosen based on clinical interest, are shown in Table 4. Of the variables, the presence of PA in sputum (HR, 2.93; 95% CI, 1.35–6.37; P = 0.007), purulent sputum production (HR, 1.51; 95% CI, 1.08–2.11; P = 0.015), onset of symptoms (HR, 1.03; 95% CI, 1.02–1.05; P < 0.001) and length of hospitalization (HR, 1.05; 95% CI, 1.01-1.08; P = 0.012) were independently associated with the presence of bronchiectasis in patients with COPD.

The differential characteristics of the group who survived follow-up (n = 333) and the group who died (n = 75) are shown in Table 5. The patients with COPD who died were older and presented with a longer duration of symptoms, a higher prevalence of bronchiectasis, a greater number of positive cultures of PPM, and a lower peripheral albumin concentration.

### Multivariate Survival Analysis

**Figure 2:**
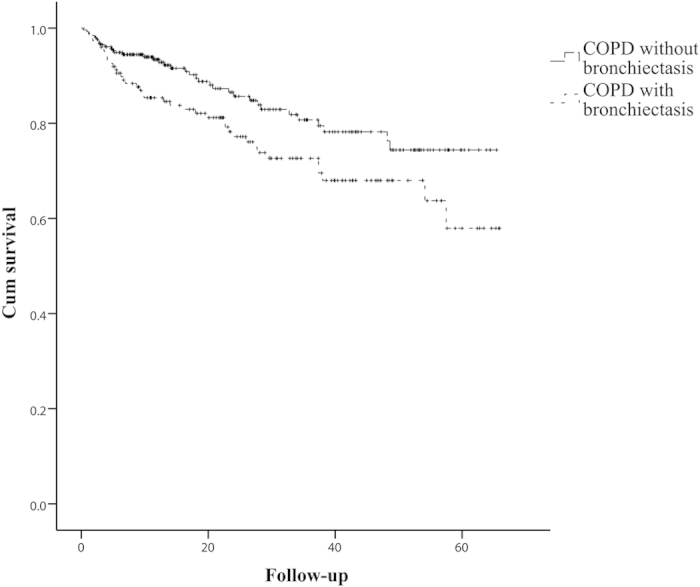
The Kaplan-Meier survival curves for patients with COPD and bronchiectasis (n = 150; 39 deaths) and for patients with COPD but without bronchiectasis (n = 258; 36 deaths). Patients with COPD with bronchiectasis had a lower survival rate than patients with COPD without bronchiectasis. There was a significant difference between the two groups (log-rank test, 5.08; P = 0.024).

Kaplan-Meier survival curves for the patients with COPD with bronchiectasis (n = 150; 39 deaths) and the patients with COPD without bronchiectasis (n = 258; 36 deaths) are shown in Fig. 2. Of all of the patients, 408 fully responded to follow-up. There was a significant difference between the two groups (log-rank test, 5.08; P = 0.024). Table 6 shows the unadjusted and fully adjusted Cox regression analysis. The risk of all-cause mortality in patients with COPD with bronchiectasis was higher than in patients with COPD without bronchiectasis(unadjusted HR, 1.68; 95% CI, 1.06-2.64; P = 0.026). In the fully adjusted model, the risk remained significantly higher in patients with COPD with bronchiectasis than in patients with COPD without bronchiectasis (fully adjusted HR, 1.77; 95% CI, 1.02-3.08; P = 0.043). Age and the isolation of PPM also had an independent adverse prognostic value in the fully adjusted model.

## Discussion

Based on our results, the isolation of PA in sputum, purulent sputum production, and longer time since the onset of symptoms were independently associated with bronchiectasis. Our study also found several factors in addition to those already known to be associated with an increased risk of all-cause mortality in patients with COPD, such as decreased pulmonary function and the subsequent complications. Specifically, the presence of bronchiectasis, the isolation of PPM, and the patient’s age were associated with an increased risk in all-cause mortality in patients with COPD.

The prevalence of bronchiectasis in patients with COPD in this study was 34.7%. Of these patients, 82.3% had cylindrical bronchiectasis. These findings are similar to the percentages reported in previous studies^9^,^10^,^26^. According to King P^27^, a deficiency in host defense combined with bacterial infection enables microbial colonization of the airways, which results in chronic inflammation and lung damage. Although no studies have demonstrated a causal association between COPD and bronchiectasis, the present study found that bronchiectasis is a likely clinical outcome among patients with COPD and the presence of bacterium in the sputum.

Patients with COPD and bronchiectasis were more likely to be female (52.6% vs. 31.5%, P < 0.001). The group of patients who had COPD without bronchiectasis was more likely to have a history of smoking (79.3% vs. 57.9%, P < 0.001) and a higher smoking index (678.1 vs. 430.9, P < 0.001). This correlation can be explained by the fact that the majority of subjects included in the study were male (84.9%). Patients with bronchiectasis had a longer duration of symptoms (14.8 y vs. 10.4 y, P < 0.001), a longer length of hospitalization (11.5 d vs. 10.2 d, P < 0.001) and more frequent purulent sputum production (52.4% vs. 41.5%, P = 0.002). These variables are associated with the structural damage caused by bronchiectasis. Patients with a history of nasosinusitis or tuberculosis were more likely to have bronchiectasis^28^. However, after adjusting for confounding variables, previous nasosinusitis was not associated with the presence of bronchiectasis. Moreover, mycobacterium tuberculosis is highly prevalent in the Chinese population; thus, it may be a confounding factor for bronchiectasis.

The most important factor associated with the presence of bronchiectasis was the isolation of PA from at least one sputum sample (HR, 2.93; 95%CI, 1.35-6.37; P = 0.007). PA was the most frequently isolated bacterium. The isolation of PA was significantly different between patients with COPD with bronchiectasis and patients with COPD without bronchiectasis (P < 0.001). Similar to the findings of previous studies, the isolation of PA was associated with the presence of bronchiectasis in this study^29^. When we assessed PA as a biomarker of bronchiectasis for COPD patients, we found that PA had a weak sensitivity of 10.5% but a strong specificity of 97.7%. Thus, this variable may help physicians to make a preliminary assessment of bronchiectasis for subsequent treatment. However, additional studies with a larger sample size are needed to confirm this finding.

In the patients with bronchiectasis, those who had cylindrical bronchiectasis presented with a higher level of PO_2_ than patients who had other types of bronchiectasis. However, there was no significant difference in PO_2_ level between patients with cystic bronchiectasis and patients with mixed bronchiectasis (P = 0.713). Habesoglu found that the clinical picture and the deterioration of the pulmonary function test might be more severe in patients with cystic bronchiectasis^30^. The discrepancy with our study can be explained by the fact that, in our study, most patients with COPD exacerbations were required to receive medical treatment, and the PO_2_ level was measured after the effect of drugs in some of these patients. Another explanation may be that the type of bronchiectasis was incorrectly classified.

Previous studies have shown that hyperglycemia during hospitalization is associated with poor outcomes in patients with COPD, which prolongs the length of stay and increases the risk of death in patients with acute exacerbated COPD^19^,^20^,^21^,^22^. However, in the present study, hyperglycemia did not have any significant predictive value for mortality in the fully adjusted survival analysis (P = 0.519). This finding can be explained by the fact that some of the patients had comorbidities that elevate blood glucose level (e.g., pheochromocytoma, hyperthyroidism, transient hyperglycemia). Further studies should be performed to assess this relationship.

Miguel-Angel *et al.* found that patients with COPD and bronchiectasis have increased bronchial inflammation; longer, more severe, and more frequent exacerbations; more PPM in the bronchial mucosa; and worse lung function^10^,^11^,^12^. Because these variables are associated with an increased risk of death in patients with COPD, the presence of bronchiectasis may also have a prognostic value these patients. The univariate analysis found that there was a significant difference in several of the variables between the patients who died during follow-up and those who survived. Thus, these variables have a prognostic value in patients who have COPD. After fully adjusting for covariates, patient age, the isolation of PPM and the presence of bronchiectasis were the only independent risk factors of all-cause mortality in patients with COPD in this study.

We found that the presence of PPM in the sputum was not significantly correlated with the presence of bronchiectasis (r = 0.07, P = 0.17), which is contrary to the findings of a previous study^12^. There may be several explanations for this discrepancy. One explanation may be that the small sample size in the present study provides insufficient statistical power. Another explanation may be that missing data on the presence of microorganisms in the sputum of some patients may have affected our results. Despite these inconsistent finding, we cannot rule out the association between PPM and bronchiectasis. The isolation of PPM from sputum samples can include PPM from a recent infection. PPM from a recent infection would be an indicator of the exacerbation of bronchiectasis, which was associated with an increase in all-cause mortality in patients with COPD in the previous study^31^,^32^,^33^,^34^. PPM includes bacterium and fungus, which were associated with the prognosis of patients who had COPD in our study. After fully adjusting for covariates, patients who had PPM detected in their sputum were 2.35 times more likely to die than patients who did not have microorganisms in their sputum culture, independent of other variables. Larger studies are needed to confirm the role of PPM in the relationship between COPD and bronchiectasis.

After adjusting for covariates, patients who had bronchiectasis were 1.77 times more likely to die than those who did not have bronchiectasis in our study. This result is similar to the findings of a previous study^12^. As a prognostic factor in patients who have COPD, bronchiectasis could help physicians to establish early treatment programs to improve the prognosis of patients with COPD. The main limitation of our study is that we recruited inpatients who were admitted with COPD; thus, these patients were more likely to have severe clinical outcomes and complications. Several factors that have been shown to be associated with all-cause mortality in patients with COPD, such as the modified Medical Research Council dyspnea scale (mMRC), the pulmonary function test and COPD exacerbation were not included in this retrospective study. However, because the comparisons between the groups were at the same stage, the results of this study still have considerable clinical value. A similar prospective study with a large sample size of stable COPD patients should be conducted in the future.

## Conclusion

Our results suggest that the duration of symptoms, the production of purulent sputum, the length of hospitalization and the isolation of PA from sputum samples were associated with the presence of bronchiectasis in patients with COPD. The patient’s age, the isolation of PPM in sputum and the presence of bronchiectasis were independent risk factors of all-cause mortality in the survival analysis of patients with COPD. When bronchiectasis is detected in older patients who have been diagnosed with COPD and whose sputum culture contains PPM, their prognosis is poorer than that of other patients. This finding emphasizes the importance of early diagnosis and treatment in patients with COPD and bronchiectasis. However, further studies are needed to demonstrate the connection between bronchiectasis and the prognosis of COPD.

## Additional Information

**How to cite this article**: Mao, B. *et al.* The existence of bronchiectasis predicts worse prognosis in patients with COPD. *Sci. Rep.* **5**, 10961; doi: 10.1038/srep10961 (2015).

## Figures and Tables

**Table 1:** Baseline and clinical characteristics of subjects with COPD, with and without bronchiectasis.

**Parameter**	**Whole Group**	**COPD with Bronchiectasis**	**COPD without Bronchiectasis**	**P**
Subjects, No. (%)	896	311 (34.7%)	585 (65.3%)	—
Sex, M/F, No.	761/135	240/71	521/64	**<0.001**
Age, y	66.2 (9.6)	66.9 (10.3)	65.8 (9.2)	**0.027**
Smoking history, No. (%)	644 (71.9%)	180 (57.9%)	464 (79.3%)	**<0.001**
Smoking index	592.3 (559.6)	430.9 (499.4)	678.1 (571.2)	**<0.001**
Body mass index, kg/m^2^	21.4(3.8)	21.1(3.8)	21.7(3.8)	**0.042**
Onset of symptoms, y	11.9 (11.1)	14.8 (13.3)	10.4 (9.4)	**<0.001**
Daily sputum production, No. (%)	706 (78.8%)	251 (80.7%)	455 (77.8%)	0.345
Purulent sputum, No. (%)	406 (45.3%)	163 (52.4%)	243 (41.5%)	**0.002**
Wheezing, No. (%)	850 (94.9%)	295 (94.9%)	555 (94.9%)	1
Previous nasosinusitis, No. (%)	16 (1.8%)	10 (3.2%)	6 (1.0%)	**0.03**
Previous pneumonia, No. (%)	9 (1.0%)	3 (1.0%)	6 (1.0%)	0.346
Previous tuberculosis, No. (%)	108 (12.1%)	62 (19.9%)	46 (7.9%)	**<0.001**
PO2, mmHg	79.6 (21.2)	81.4 (23.7)	78.6 (19.7)	0.352
PCO2, mmHg	44 (10.9)	43.7 (10.5)	44.1 (11.1)	0.667
Hypercapnia, No. (%)	288(32.1%)	109(35%)	179(30.6%)	0.175
Albumin, mg/dL	38.4 (4.6)	38 (4.4)	38.7 (4.7)	0.067
CRP, IU/mL	17 (33.7)	17.6 (28.2)	16.7 (36.2)	**0.001**
Blood glucose, mmol/L	5.7 (1.9)	5.7 (2)	5.8 (1.8)	0.152
Hb, g/L	135.8 (17.7)	134.2 (16.7)	136.7 (18.2)	**0.022**
ESR,mm/H	28.4 (24.7)	34.4 (26.7)	25.2 (22.9)	**<0.001**
Length of hospitalization, d	10.7 (5.3)	11.5 (5.8)	10.2 (5.1)	**<0.001**

Data are presented as n (%) or mean ±SD unless otherwise stated. CRP: C-reactive protein; Hb: hemoglobin; ESR: erythrocyte sedimentation rate.

**Table 2:** Microbiologic characteristics of subjects with COPD, with and without bronchiectasis.

**Parameter**	**Whole Group**	**COPD with Bronchiectasis**	**COPD without Bronchiectasis**	**P**
Subjects, No. (%)	726	257	469	—
Mycobacterium tuberculosis detected, No. (%)	11 (1.5%)	3 (1.2%)	8 (1.7%)	0.802
NTM detected, No. (%)	8 (1.1%)	2 (0.8%)	6 (1.3%)	0.805
Patients with at least one PPM isolate, No. (%)	372 (51.2%)	146 (56.8%)	226 (48.2%)	**0.026**
Bacteriologic	127 (17.5%)	60 (23.3%)	67 (14.3%)	**0.002**
Pseudomonas aeruginosa isolates, No. (%)	38 (5.2%)	27 (10.5%)	11 (2.3%)	**<0.001**
Klebsiella pneumoniae isolates, No. (%)	26 (3.6%)	5 (1.9%)	21 (4.5%)	0.079
Escherichia coli isolates, No. (%)	4 (0.6%)	3 (1.2%)	1 (0.2%)	0.256
Baumannii isolates, No. (%)	22 (3%)	12 (4.7%)	10 (2.1%)	0.057
Enterobacter cloacae isolates, No. (%)	10 (1.4%)	4 (1.6%)	6 (1.3%)	1
Mycological	288 (39.7%)	112 (43.6%)	176 (37.5%)	0.111
Aspergillus isolates, No. (%)	40 (5.5%)	17 (6.6%)	23 (4.9%)	0.334
Monilia albicans isolates, No. (%)	247 (34%)	98 (38.1%)	147 (31.3%)	0.058

Data are presented as n (%) or mean ±SD unless otherwise stated. NTM: nontuberculosis mycobacteria; PPM: potentially pathogenic microorganisms.

**Table 3:** Analytic characteristics of subjects with COPD and bronchiectasis according to the type of bronchiectasis.

	**Type of Bronchiectasis**			
**Parameter**	**Cylindrical**	**Cystic**	**Mixed**	**P**
Subjects, No. (%)	256 (82.3%)	35 (11.3%)	20 (6.4%)	–
Sex, M/F, No.	206/50	23/12	11/9	**0.008**
Age, y	67.4 (10)	64.3 (12)	64.1 (10.6)	0.115
PO2, mmHg	83.4 (24.3)	72.6 (18.4)	70 (18.2)	**0.004**
PCO2, mmHg	43.2 (10.8)	46.4 (9.2)	46.6 (7.8)	0.114
Albumin, mg/dL	38.2 (4.3)	37.8 (4.8)	36.9 (5.5)	0.397
CRP, IU/mL	17.9 (29.4)	14.6 (20.3)	18.6 (25.7)	0.827
Blood glucose, mmol/L	5.7 (2)	5.7 (2)	5 (0.8)	0.246
Hb, g/L	133.8 (17.2)	134.5 (15.7)	138.5 (10.3)	0.487
ESR, mm/H	34.2 (27.3)	33.8 (24.9)	38.2 (21.8)	0.806
Length of hospitalization, d	11.6 (6.1)	11.7 (4.2)	10.4 (3.7)	0.641

Data are presented as n (%) or mean ±SD unless otherwise stated.

**Table 4:** Variables associated with the presence of bronchiectasis in a logistic regression model.

	**Unadjusted**		**Fully Adjusted**	
**Variables**	**HR (95% CI)**	**P**	**HR (95% CI)**	**P**
Onset of symptoms	1.04 (1.02–1.05)	<0.001	**1.03** (**1.02–1.05)**	**<0.001**
Purulent sputum	1.55 (1.18–2.04)	0.002	**1.51** (**1.08–2.11)**	**0.015**
Previous nasosinusitis	3.21 (1.15–8.91)	0.025	2.98 (0.97–9.20)	0.057
Isolation of PA	4.89 (2.38–10.03)	<0.001	**2.93** (**1.35–6.37)**	**0.007**
Length of hospitalization	1.05 (1.02–1.08)	0.001	**1.05** (**1.01–1.08)**	**0.012**

PA: Pseudomonas aeruginosa; HR: hazard ratio; CI: confidence interval. Variables were adjusted for age, sex, smoking history, smoking index, body mass index, onset of symptoms, purulent sputum, CRP, Hb, ESR, and length of hospitalization.

**Table 5:** Baseline and clinical characteristics of patients according to their vital status at the end of the study.

**Parameter**	**Whole Group**	**Died**	**Survived**	**P**
Subjects, n	408	75	333	–
Age, y	67.4 (9.0)	73.1 (8.2)	66.2 (8.5)	**<0.001**
Sex, M/F, No.	348/60	64/11	284/49	0.992
Onset of symptoms, y	12.4 (11.6)	14.2 (12.1)	11.7 (10.9)	**0.028**
Pack-years smoked	29.6 (27.2)	35 (29.1)	29.1 (26.6)	0.123
Body mass index, kg/m^2^	21.5(3.8)	21.0(3.6)	21.6(3.8)	0.216
Hb, g/L	135.6 (17.4)	133.2 (23.7)	136.4 (15.3)	**0.018**
CRP, IU/mL	17.4 (28.8)	23.6 (43.9)	16.0 (24.2)	**0.017**
Albumin, mg/dL	38.2 (4.4)	37.0 (4.2)	38.3 (4.4)	**0.002**
Blood glucose, mmol/L	5.8 (1.9)	6.0 (2.6)	5.7 (1.7)	0.772
Length of hospitalization, d	11 (5.9)	12.5 (4.9)	10.5 (4.7)	**0.005**
Isolation of PPM, No.	184 (54.6%)	45 (75%)	139 (50.2%)	**<0.001**
Bronchiectasis	150 (36.8%)	39 (52%)	111 (33.3%)	**0.002**

Data are presented as n (%) or mean ±SD unless otherwise stated.

**Table 6:** Variables associated with all-cause mortality in patients with COPD in a Cox proportional hazard regression model.

	**Unadjusted**		**Fully adjusted**	
**Variables**	**HR (95% CI)**	**P**	**HR (95% CI)**	**P**
Age	1.08 (1.06–1.11)	**<**0.001	**1.09** (**1.05–1.13)**	**<0.001**
Hb	0.99 (0.97–1.0)	0.027	1.01 (0.99–1.03)	0.236
CRP	1.01 (1.0–1.02)	0.005	1.01 (1.0–1.02)	0.079
Albumin	0.91 (0.86–0.96)	**<**0.001	0.98 (0.91–1.05)	0.578
Isolation of PPM	2.62 (1.46–4.70)	0.001	**2.35** (**1.22–4.52)**	**0.011**
Bronchiectasis	1.68 (1.06–2.64)	0.026	**1.77** (**1.02–3.08)**	**0.043**

Variables were adjusted for age, onset of symptoms, Hb, CRP, albumin, and length of hospitalization.
